# Chlorination of Phenethyl Isothiocyanate Potentiates Cytotoxicity and Apoptosis in Multidrug-Resistant Leukemia Cells

**DOI:** 10.3390/ijms27135869

**Published:** 2026-06-29

**Authors:** Alberto Yoldi Vergara, Anna Bertova, Szilvia Kontar, Martina Ksinanova, Kristina Simonicova, Martin Simkovic, Zdena Sulova, Albert Breier, Denisa Imrichova

**Affiliations:** 1 Institute of Molecular Physiology and Genetics, Centre of Biosciences, Slovak Academy of Sciences, Dúbravská Cesta 9, 841 04 Bratislava, Slovakia; umfgyolv@savba.sk (A.Y.V.); anna.bertova@savba.sk (A.B.); szilvia.kontar@savba.sk (S.K.); martina.ksinanova@savba.sk (M.K.); kristina.simonicova@savba.sk (K.S.); zdena.sulova@savba.sk (Z.S.); 2 Institute of Biochemistry and Microbiology, Faculty of Chemical and Food Technology, Slovak University of Technology in Bratislava, Radlinského 9, 812 37 Bratislava, Slovakia; martin.simkovic@stuba.sk

**Keywords:** isothiocyanate (ITC), phenethyl isothiocyanate (PEITC), 2-(4-Chlorophenethyl) isothiocyanate (Cl-PEITC), oxidative stress, apoptosis, autophagy, leukemia

## Abstract

In medicinal chemistry, halogen substitution is often used to enhance the biological activity of anticancer compounds. Phenethyl isothiocyanate (PEITC), a natural compound found in cruciferous vegetables, exhibits anti-cancer activity by modulating oxidative stress and apoptosis-related pathways. This study compared the effects of PEITC and its chlorinated derivative, Cl-PEITC, on human leukemia cell lines, including multidrug-resistant (MDR) variants that overexpress P-glycoprotein (P-gp). We evaluated cell viability, apoptosis, reactive oxygen species (ROS) production, the modulation of the NRF2/KEAP1 signaling pathway, NF-κB p65 protein expression, DNA fragmentation, and autophagy in SKM-1, MOLM-13 and their MDR variants SKM/VCR and MOLM/VCR cells. Cl-PEITC exhibited stronger antiproliferative and cytotoxic effects than PEITC in all tested cell lines and maintained similar activity in P-gp-positive resistant cells. In contrast, resistant sublines showed reduced sensitivity to PEITC. Cl-PEITC induced higher ROS production and enhanced apoptosis, accompanied by the activation of caspases-3, -8, and -9 and PARP1 cleavage. It also caused more pronounced DNA fragmentation. Both PEITC and Cl-PEITC modulated autophagy-related markers, as demonstrated by increased LC3-II/LC3-I conversion and decreased p62 protein levels. In addition, these compounds modulated NRF2/KEAP1 and reduced NF-κB p65 expression in a concentration-dependent manner. These findings suggest that the chlorination of PEITC enhances its antileukemic activity and could retain its efficacy against P-gp-associated MDR.

## 1. Introduction

Neoplastic diseases remain the most common therapeutic area for newly approved drugs. According to the Center for Drug Evaluation and Research, a division of the FDA, Mullard [1] reported that 35% of drugs approved in 2025 were indicated for the treatment of malignancies. This represented an increase from the five-year rolling average of 29%. In addition to new chemical entities derived from organic and bioorganic synthesis, research continues to focus on natural substances—bioactive compounds from plants, microbes, and other organisms—that can significantly impact cellular homeostasis and fundamental regulatory processes [2].

The search for bioactive natural products from plants and microorganisms (both prokaryotic and eukaryotic) has significantly contributed to the discovery and development of pharmaceuticals important for global healthcare. However, numerous studies have demonstrated that neoplastically transformed cells can develop drug resistance through multiple mechanisms, thereby limiting the efficacy of current chemotherapeutic agents across various cancer types [3]. This underscores the need to identify additional safe and effective antiproliferative agents, including plant-derived compounds. Early studies by Horáková et al. [4] demonstrated that isothiocyanates (ITCs), predominantly found in cruciferous vegetables, exhibit antitumor activity. More recent research has shown that these molecules modulate a broad range of regulatory and metabolic pathways [5].

Over time, our knowledge of the potential applications of ITCs in preventing and treating various malignancies has grown. Although the literature directly focusing on leukemia is comparatively limited, several noteworthy findings have been reported. One review article discusses the anticarcinogenic properties of sulforaphane (SFN), the need for doses exceeding those achievable through diet, and the challenges in evaluating SFN as an adjunct to standard therapy in hematological malignancies [6]. We have previously demonstrated that allyl isothiocyanate (AITC) and SFN inhibit the growth of mouse lymphoblastic leukemia cells, regardless of P-glycoprotein (P-gp) expression [7,8]. P-gp belongs to the ABC transporter family and mediates the efflux of various structurally unrelated drugs from cells [9]. First identified in 1976, P-gp was the first ABC transporter to be discovered and remains one of the most extensively studied mediators of MDR [10]. In a subsequent study, we demonstrated the effectiveness of SFN and benzyl isothiocyanate (BITC) in suppressing the proliferation of the human acute myeloid leukemia cell lines SKM-1 and SKM-1/VCR. The latter is P-gp-positive and drug-resistant [11]. The more lipophilic BITC exhibited an IC_50_ below 5 µM, whereas the more hydrophobic SFN showed an IC_50_ below 8 µM. Both compounds exerted significant cytotoxic effects; however, BITC demonstrated a greater capacity to induce apoptosis, including in multidrug-resistant cells. In contrast, sulforaphane preferentially induced autophagy in a LC3-dependent manner.

In addition to SFN and AITC or BITC, phenethyl isothiocyanate (PEITC) is renowned for its diverse pharmacological effects [12]. This compound is released enzymatically from gluconasturtiin, a naturally occurring glucosinolate found in watercress [13]. As early as 1968, Horáková et al. reported that phenylisothiocyanate exerted a cytotoxic effect more than three times higher than that of AITC on HeLa cells [14]. The introduction of a chlorine substituent at the para position of the benzene ring produced an ITC derivative that was over four times more cytotoxic than AITC. Halogen substitution has become an important strategy in medicinal chemistry because halogen atoms frequently improve the biological and pharmacokinetic properties of therapeutic compounds [15,16]. Recent analyses of FDA-approved drugs indicate that halogen-containing molecules represent a substantial proportion of newly developed pharmaceuticals, including agents used in oncology [17].

PEITC has been widely studied for its anticancer properties and has been shown to induce cytotoxicity in various tumor models by activating apoptotic signaling pathways, including caspase activation and PARP cleavage. Additionally, PEITC has been reported to modulate key cellular survival pathways, thereby contributing to its overall antitumor activity. In acute myeloid leukemia (AML) models, PEITC has demonstrated significant antileukemic effects in both in vitro and in vivo settings. These effects include inducing apoptosis in leukemia cells and suppressing tumor growth in xenograft models. These findings support the potential relevance of PEITC in the treatment of hematological malignancies [18].

Motivated by these findings, we investigated the effects of PEITC and its p-chlorophenethyl derivative (Cl-PEITC) on SKM-1 and MOLM-13 acute myeloid leukemia cells, as well as on their P-gp-positive, drug-resistant variants, SKM/VCR and MOLM/VCR. The presence of chlorine on the aromatic ring is associated with increased molecular polarizability and enhanced lipophilicity [19]. An increase in lipophilicity may facilitate penetration into hydrophobic cellular structures and biological membranes. The two CH_2_ groups of the ethyl linker act as a spacer, reducing direct electronic communication between the aromatic π-electron system and the isothiocyanate moiety. Therefore, substitution on the aromatic ring is only expected to have a limited direct electronic effect on the intrinsic reactivity of the ITC group. Nevertheless, increased polarizability of the chlorinated derivative may enhance its interactions with nucleophilic biomolecules, such as thiol (–SH), amino (–NH_2_), or hydroxyl (–OH) groups. The increase in lipophilicity and molecular polarizability associated with para-chloro substitution on aromatic rings is well documented and represents a common strategy in medicinal chemistry [20]. These physicochemical modifications may facilitate membrane penetration and enhance interactions with biological targets, potentially improving the efficacy and biological activity of compounds in drug-resistant cells [21].

## 2. Results

### 2.1. Effect of PEITC and Cl-PEITC on Cell Proliferation and Viability

**Figure 1:**
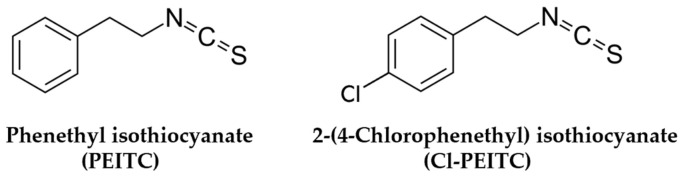
Chemical structures of PEITC and Cl-PEITC.

The chemical structures of PEITC and Cl-PEITC are shown in Figure 1. Introducing a chlorine atom in the para position relative to the ethyl ITC group on the benzene ring increases the compound’s molecular weight, lipophilicity, and polarizability. Meanwhile, its ability to form hydrogen bonds and its polar surface area remain essentially unchanged. Consequently, the chlorinated derivative is expected to exhibit improved passive membrane permeability and stronger hydrophobic interactions with biological macromolecules. Substituting hydrogen on the aromatic skeleton with chlorine is a common medicinal chemistry strategy to enhance cellular uptake, metabolic stability, and binding to target molecules in small-molecule drug candidates [22].

**Figure 2:**
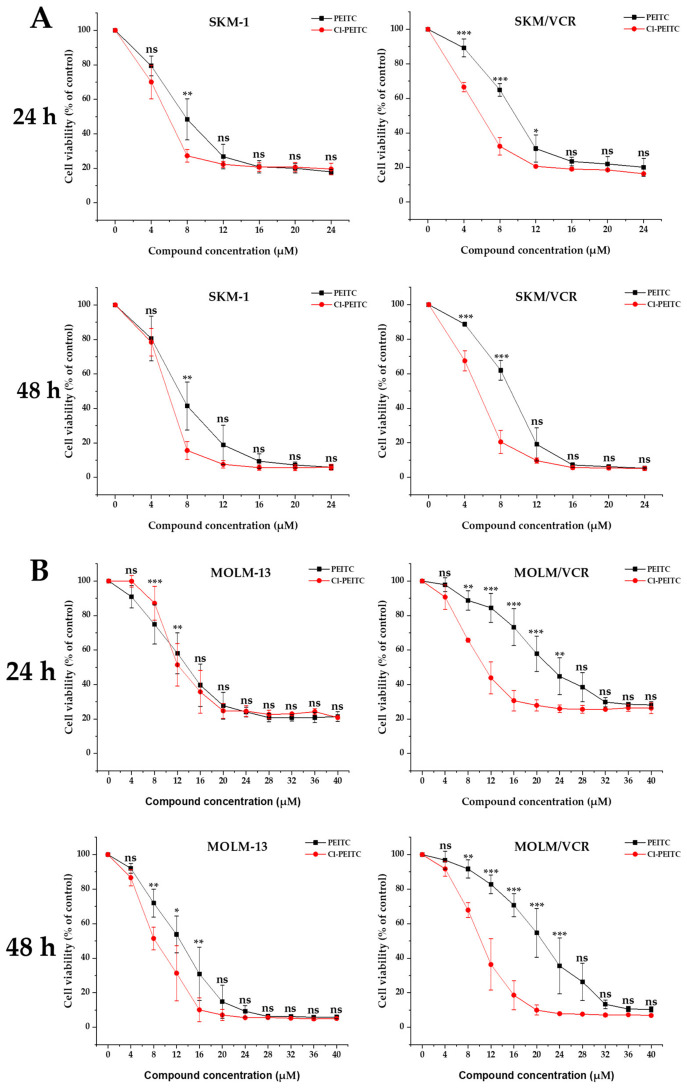
Comparison of the cytotoxic effects of PEITC or Cl-PEITC on SKM-1 and MOLM-13 cells and their P-glycoprotein-positive, vincristine-resistant variants (SKM/VCR and MOLM/VCR). Dose-dependent inhibition of cell proliferation by PEITC or Cl-PEITC in (**A**) SKM-1, SKM/VCR, and (**B**) MOLM-13 and MOLM/VCR cell lines. The cells were exposed to increasing concentrations of PEITC or Cl-PEITC for 24 and 48 h. Data were analyzed by two-way ANOVA with Tukey’s post hoc multiple comparison test (ns—not significant; * *p* < 0.05, ** *p* < 0.01, and *** *p* < 0.001).

In the first phase of this study, we compared the antiproliferative potential of these two ITCs in the human leukemia cell lines SKM-1 and SKM/VCR (Figure 2A) and MOLM-13 and MOLM/VCR (Figure 2B). We used a CASY model TT cell counter to enable precise cell counting and to determine cell size distribution, aggregation, and viability. The cells were exposed to increasing concentrations of PEITC (0, 4, 8, 12, 16, 20, and 24 µM) or Cl-PEITC (0, 4, 8, 12, 16, 20, 24, 28, 32, 36, and 40 µM) for 24 and 48 h. The growth of all cell lines gradually decreased in a concentration-dependent manner. Two-way ANOVA revealed significant effects of concentration and treatment, as well as their interaction, at both time points. This indicates that the cytotoxic response depends on the concentration used and the compound.

Overall, Cl-PEITC showed greater cytotoxicity than PEITC, though the difference varied across cell lines. In SKM-1 cells, both compounds produced effects that were largely comparable. Conversely, SKM/VCR and MOLM/VCR cells were more sensitive to Cl-PEITC, showing significant differences over a broader concentration range. MOLM-13 cells displayed an intermediate response, with differences between the two compounds mainly evident at intermediate concentrations.

No significant differences were observed between PEITC and Cl-PEITC at the lowest concentrations (0–4 µM) in SKM-1, MOLM-13, and MOLM/VCR cells. However, SKM/VCR cells responded differently at 4 µM. At higher concentrations, the effects of both compounds gradually converged. Consistent with these findings, IC_50_ values obtained after 24 and 48 h exposures differed only slightly (Table 1), indicating that most of the antiproliferative activity occurred within the first 24 h.

Overall, these results demonstrate that Cl-PEITC has stronger antiproliferative effects than PEITC, especially in vincristine-resistant leukemia cell lines.

### 2.2. Impact of PEITC and Cl-PEITC Treatment on the Metabolic Activity of Leukemia Cells

We assessed cellular metabolic activity using an MTS assay. Although tetrazolium-based assays (e.g., MTT and MTS) reflect the activity of NAD(P)H-dependent dehydrogenases in the cytosol and mitochondria, they are commonly used in the literature as proxies for cell viability and cytotoxicity. This may lead to misinterpretation of the results [23,24]. Depending on the type of sublethal damage, cells may respond by activating protective pathways associated with increased metabolic activity. For example, γ-irradiation has been shown to stimulate mitochondrial dehydrogenase activity, resulting in discrepancies between the MTS signal and the actual number of viable cells [25]. To account for this effect, we evaluated cell responses using both an MTS assay and direct cell counting. Such discrepancies were observed in our experiments with sulforaphane (SFN) treatment of mouse lymphoblastic leukemia cells, where the MTS signal did not proportionally reflect the number of viable cells [7].

**Figure 3:**
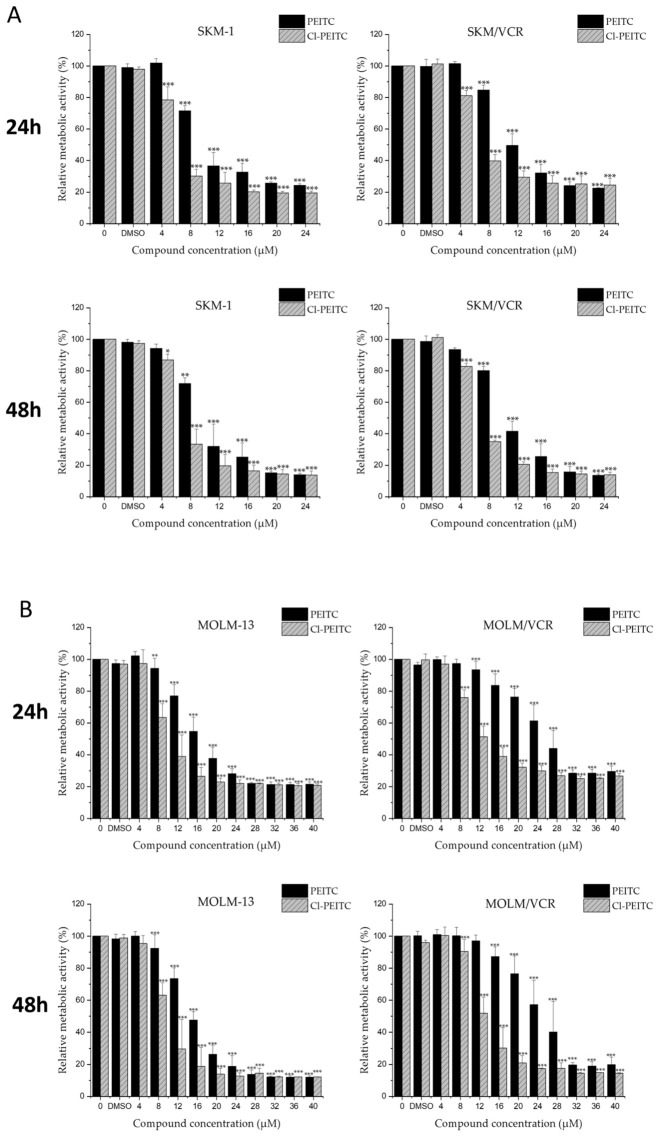
Cell viability according to the MTS test. (**A**) SKM-1 and SKM/VCR cells after 24 and 48 h of incubation with PEITC or Cl-PEITC at concentrations ranging from 0 to 24 µM. (**B**) MOLM-13 and MOLM/VCR cells after 24 and 48 h of incubation with PEITC or Cl-PEITC at concentrations ranging from 0 to 40 µM. The results are expressed as the mean ± SD from three independent experiments performed in six replicates. One-way ANOVA followed by Tukey’s post hoc test (* *p* < 0.05, ** *p* < 0.01, *** *p* < 0.001 vs. control cells) was used to assess statistical significance.

Figure 3 shows the effects of PEITC and Cl-PEITC on the metabolic activity of the tested leukemia cell lines. As shown, Cl-PEITC exhibited higher cytotoxic potential than PEITC in this assay. After 24 h of treatment with 4 µM Cl-PEITC, a statistically significant decrease in cell viability was observed in SKM-1 and SKM/VCR cells (Figure 3A). In contrast, a significant effect was observed in MOLM-13 and MOLM/VCR cells at 8 µM Cl-PEITC (Figure 3B).

Cells exhibiting a P-gp-associated MDR phenotype showed reduced sensitivity to PEITC, as indicated by the IC_50_ values (Table 1), compared to their parental counterparts. After 24 and 48 h, the MOLM/VCR subline exhibited increased resistance to PEITC compared to the MOLM-13 parental cells (MOLM-13: 14.85 µM vs. MOLM/VCR: 23.03 µM and MOLM-13: 14.72 µM vs. MOLM/VCR: 23.57 µM, respectively). In contrast, minor differences were observed between P-gp-positive and P-gp-negative cells following Cl-PEITC treatment (8.64 µM vs. 9.99 µM and 8.72 µM vs. 11.61 µM at 24 and 48 h, respectively). These results indicate that Cl-PEITC is more effective than the non-chlorinated derivative in reducing cell viability and is active irrespective of P-gp expression.

When comparing the efficacy of the two ITCs using direct cell counting and the MTS assay, higher IC_50_ values were consistently obtained using the MTS assay. This difference may be attributed to the ability of ITCs to interfere with cellular redox balance [26], which can enhance the reduction in MTS and thereby increase the measured signal, leading to an overestimation of cell viability. However, no deviation from proportionality was observed between the MTS signal and the number of viable cells for PEITC and Cl-PEITC. The correlation between the MTS signal and the number of viable cells was strong and statistically significant (with regression line: MTS signal = 0.078 + 1.213 × number of viable cells, with a correlation coefficient of 0.993 (Supplementary Figure S1). The higher IC_50_ values obtained with the MTS assay compared to direct cell counting are consistent with the positive intercept of the regression equation and a slope greater than 1.

We calculated a resistance index (RI), defined as the ratio of the IC_50_ of resistant cell lines to that of their sensitive counterparts, to assess the degree of resistance of the P-gp-positive cell lines SKM/VCR and MOLM/VCR to PEITC and Cl-PEITC. We determined RI values for both ITCs using two methods: direct cell counting (CASY TT Count) and the MTS assay (Table 2). Comparable results were obtained with both methods. However, direct cell counting revealed a more pronounced decrease in sensitivity of P-gp-positive cells to PEITC compared to their P-gp-negative counterparts than was observed with the MTS assay.

For SKM/VCR cells, RI values for Cl-PEITC approached 1 after 24 h of incubation and fell below 1 after 48 h, as determined by both methods. These cells exhibited only mild resistance to PEITC, with RI values below 1.3, as determined by direct cell counting. Slightly lower RI values were obtained using the MTS assay (1.22 and 1.14 at the 24 h and 48 h time points, respectively).

In contrast, MOLM/VCR cells showed more pronounced resistance to PEITC, with RI values exceeding 1.5 at both 24 and 48 h time points. However, these cells were less resistant to Cl-PEITC than to PEITC. Overall, these results suggest that introducing a halogen substituent into the benzene ring of PEITC may retain its efficacy against multidrug-resistant cells overexpressing P-gp.

### 2.3. PEITC and Cl-PEITC Induced Apoptosis in the Leukemia Cells

**Figure 4:**
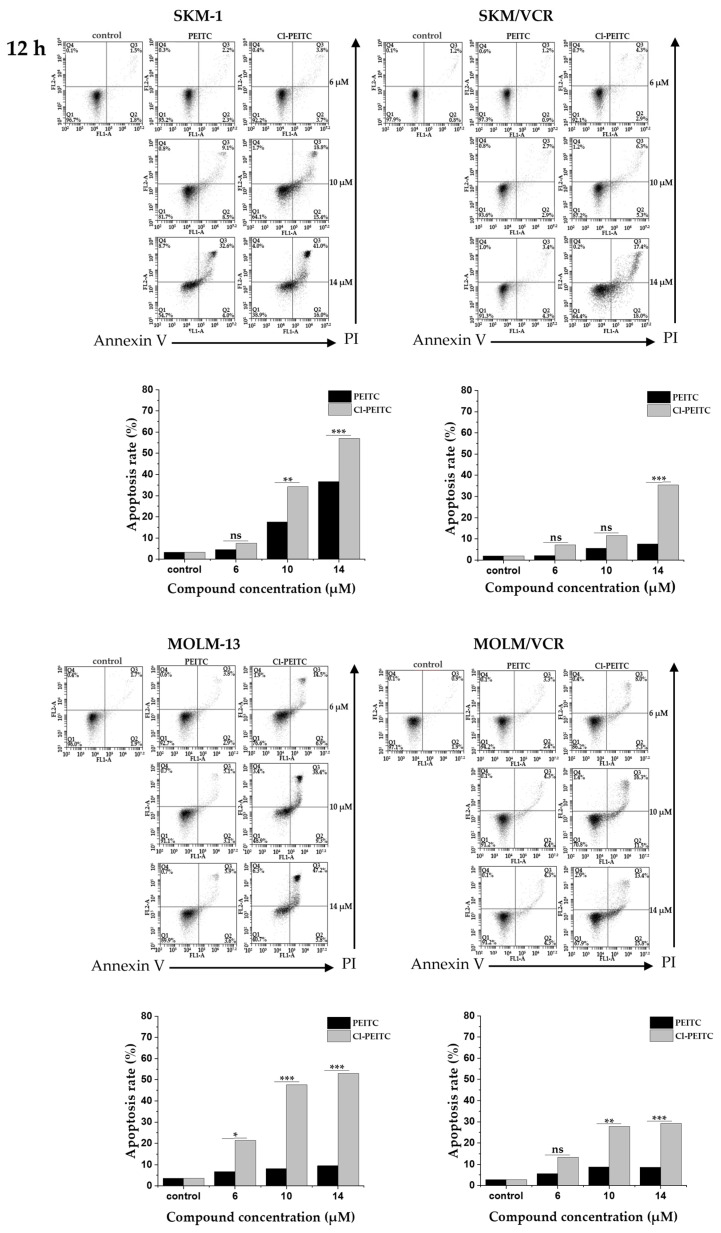
Apoptosis induced by PEITC and Cl-PEITC in AML cell lines. SKM-1 and SKM/VCR, MOLM-13 and MOLM/VCR cells were treated with PEITC or Cl-PEITC at concentrations of 6, 10, and 14 µM for 12 h. Apoptosis induction was detected using an Annexin V-FITC kit and flow cytometry, as described in the Materials and Methods section. Each panel includes a representative cytogram and a quantitative data. Statistical significance was assessed using two-way ANOVA with Tukey’s post hoc multiple comparison test, allowing pairwise evaluation of treatment concentration and compound type (PEITC vs. Cl-PEITC). Statistical significance was defined as * *p* < 0.05, ** *p* < 0.01, and *** *p* < 0.001; ns—not significant.

Apoptosis is characterized by the translocation of phosphatidylserine from the inner to the outer layer of the plasma membrane. To evaluate the pro-apoptotic effects of PEITC and Cl-PEITC on leukemia cells, we employed annexin V-FITC, a phosphatidylserine-binding dye, along with the nuclear stain propidium iodide (PI). Leukemia cell lines were treated with different concentrations of PEITC or Cl-PEITC for 3 h, 6 h (Supplementary Figure S2), and 12 h (Figure 4). The cells were then harvested and analyzed by flow cytometry to evaluate apoptosis induction. The staining results showed increased annexin V-FITC binding in treated leukemia cells compared to untreated controls. However, Cl-PEITC exhibited a significantly stronger pro-apoptotic effect than PEITC. Cl-PEITC (14 µM) induced both early apoptosis (annexin V-positive, PI-negative cells) and late apoptosis (annexin V-positive, PI-positive cells), affecting approximately 57% of SKM-1 cells, 35% of SKM/VCR cells, 53% of MOLM-13 cells, and 30% of MOLM/VCR cells. In comparison, PEITC induced apoptosis in approximately 36% of SKM-1 cells, 7.7% of SKM/VCR cells, 9.5% of MOLM-13 cells, and 8.6% of MOLM/VCR cells following a 12 h incubation period. Two-way ANOVA revealed a significant interaction between treatment concentration and compound type (PEITC vs. Cl-PEITC) in most experimental conditions, indicating that the concentration-dependent induction of apoptosis differed between the two compounds.

### 2.4. Proteolytic Activation of Caspases and Cleavage of PARP Induced by PEITC and Cl-PEITC

We investigated the activation status of the initiator caspases, caspases-8 and -9, and the executioner caspase, caspase-3, during apoptosis induced by PEITC and Cl-PEITC. The activation of caspase-3 is known to mediate the cleavage of multiple substrates, including caspase-activated DNase and poly(ADP-ribose) polymerase (PARP) [27]. PARP1, a member of the PARP family, plays a key role in the repair of single-strand DNA breaks; its cleavage is widely regarded as a hallmark of apoptosis and reflects the inactivation of DNA repair processes.

**Figure 5:**
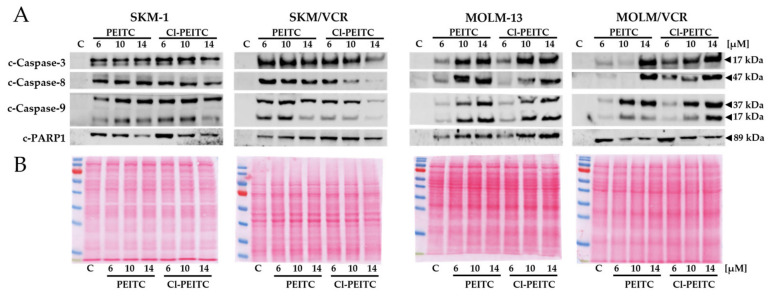
Activation of apoptotic markers following PEITC and Cl-PEITC treatment. (**A**) Whole-cell lysates from four cell lines that were treated with PEITC or Cl-PEITC at concentrations of 6, 10, and 14 µM for 12 h were analyzed by Western blot to detect cleaved caspase-3, -8, -9, and cleaved poly(ADP-ribose) polymerase (PARP). (**B**) Ponceau S staining was used as an internal loading control to verify the protein loading (60 µg/lane).

We assessed caspase activation and PARP1 cleavage in all four tested leukemic cell lines using Western blot analysis with antibodies specific to cleaved forms, aiming to determine their involvement in ITC-induced apoptosis. Consistent with the apoptosis data, treatment with PEITC and Cl-PEITC resulted in increased proteolytic activation of caspases-3, -8, and -9, accompanied by PARP1 cleavage (Figure 5). These findings indicate that both compounds trigger a cascade of molecular events leading to the activation of initiator caspases (caspases-8 and -9), executioner caspase-3, and subsequent PARP1 cleavage associated with inhibition of single-strand DNA break repair.

Notably, treatment with PEITC or Cl-PEITC affected the expression levels of commonly used housekeeping proteins, including GAPDH and β-actin (Supplementary Figure S3). A similar effect was previously observed following treatment with SFN or BITC [11]. Therefore, these proteins were considered unsuitable for normalization in Western blot analyses. Ponceau S total protein staining was used for total-protein normalization across samples and to verify equal protein loading and transfer efficiency in all Western blot analyses.

### 2.5. PEITC and Cl-PEITC Increase Intracellular ROS Levels in Sensitive Cancer Cells in a Caspase-Dependent Manner

Although the induction of oxidative stress is a key mechanism underlying the efficacy of many anticancer therapies, it can also paradoxically contribute to the transformation of normal cells into neoplastic cells [28]. In addition, oxidative stress plays a role in regulating various cellular processes, including proliferation, differentiation, and programmed cell death.

**Figure 6:**
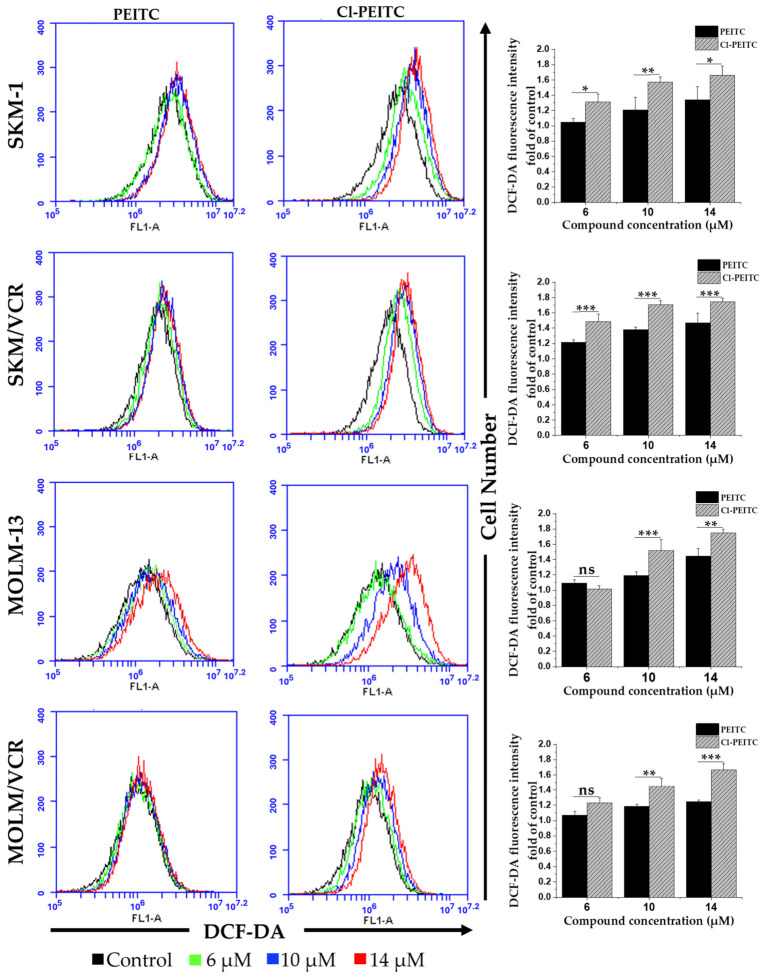
Analysis of ROS production induced by PEITC and Cl-PEITC. DCF-DA staining and flow cytometry were used to measure ROS levels in two sensitive (SKM-1 and MOLM-13) and two resistant (SKM/VCR and MOLM/VCR) leukemia cell lines. Cells were treated with PEITC or Cl-PEITC at concentrations of 6, 10, and 14 µM for 20 min. A Representative FACS histograms of ROS production illustrate the shift in the abscissa of the fluorescent cell population after treatment compared with untreated controls. Data are presented as mean ± SD of three independent experiments. Statistical significance was assessed using two-way ANOVA followed by Tukey’s post hoc multiple comparison test to evaluate the effects of compound (PEITC vs. Cl-PEITC) and concentration. Significant differences are indicated as * *p* < 0.05, ** *p* < 0.01, *** *p* < 0.00; ns—not significant.

Intracellular ROS levels in response to PEITC and Cl-PEITC were assessed in leukemia cell lines and their resistant variants using DCF-DA as a fluorescent indicator (Figure 6). Treatment with both compounds resulted in a concentration-dependent increase in ROS levels across all cell lines tested. Cl-PEITC consistently elicited higher ROS levels than PEITC, indicating a greater capacity to induce oxidative stress.

Two-way ANOVA was performed to evaluate the effects of compound type and concentration. A significant interaction between these factors was observed in MOLM-13 and MOLM/VCR cells, indicating that the difference between PEITC and Cl-PEITC varied across concentrations. In contrast, no significant interaction was detected in SKM-1 and SKM/VCR cells, suggesting that the effects of compound type and concentration were largely additive.

### 2.6. PEITC and Cl-PEITC Enhance NRF2 Protein Levels in Antioxidant Defense

The balance between the production and scavenging of ROS must be tightly regulated. Otherwise, intracellular ROS levels will increase. Endogenous ROS are continuously generated as by-products of cellular metabolism, particularly during redox reactions. In addition, cells can be exposed to exogenous pro-oxidant stimuli, including small xenobiotic molecules and ionizing radiation [29].

The antioxidant response is increasingly recognized as a context-dependent process that can both protect against tumor initiation and contribute to disease progression and therapy resistance. A key regulator of this response is the transcription factor NRF2, which controls cellular antioxidant defense by inducing the expression of numerous cytoprotective and antioxidant genes [30]. Under physiological conditions, NRF2 levels are tightly controlled by its negative regulator KEAP1, which targets NRF2 for ubiquitination and subsequent proteasomal degradation [31].

**Figure 7:**
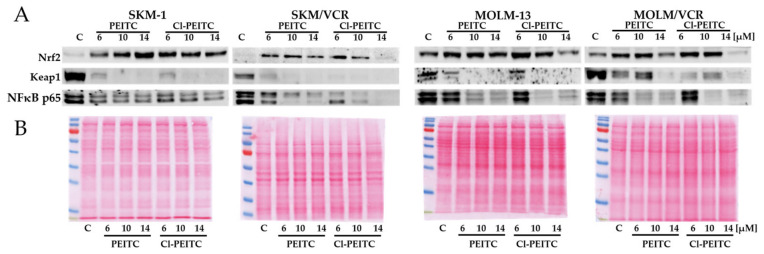
Effect of PEITC and Cl-PEITC on oxidative stress-related protein expression: NRF2, Keap1 and NFκBp65. (**A**) Proteins from four cell lines that were treated with PEITC or Cl-PEITC at concentrations of 6, 10, and 14 µM for 12 h were detected by Western blotting analyses. (**B**) Ponceau S staining was used as an internal loading control to verify the protein loading (60 µg/lane).

To compare the effects of PEITC and Cl-PEITC on the activation of the NRF2 pathway, the protein expression levels of NRF2 and KEAP1 were assessed (Figure 7). Treatment with lower concentrations of PEITC and Cl-PEITC (at 6 and 10 µM) resulted in increased NRF2 protein levels, accompanied by decreased KEAP1 expression, which suggests the activation of cytoprotective pathways. In contrast, the highest concentration tested (14 µM) suppressed NRF2 expression, particularly in P-gp-positive sublines.

Our results also showed reduced levels of NF-κB p65 (RelA) protein following treatment with PEITC and Cl-PEITC (Figure 7). NF-κB p65 typically forms a functional transcriptional heterodimer with NF-κB p50 [32]. The NF-κB p50/RelA pathway regulates genes involved in cell survival, inflammation, and redox homeostasis, and its inhibition is commonly associated with reduced expression of anti-apoptotic and cytoprotective factors [33].

Therefore, the observed decrease in NF-κB p65 following ITCs treatment is consistent with the well-established crosstalk between the NRF2 and NF-κB signaling pathways.

### 2.7. Effect of PEITC and Cl-PEITC on Expression of NRF2 Target Gene HMOX1

Heme oxygenase 1 (HO-1, which is encoded by the *HMOX1* gene) is a stress-inducible enzyme that catalyzes the degradation of heme into biliverdin, carbon monoxide (CO), and ferrous iron (Fe^2+^). *HMOX1* gene expression is predominantly regulated by the transcription factor Nrf2, and HO-1 is the rate-limiting enzyme in heme catabolism. Consequently, HO-1 plays a crucial role in mediating antioxidant, anti-inflammatory, and cytoprotective responses under conditions of oxidative stress [34].

**Figure 8:**
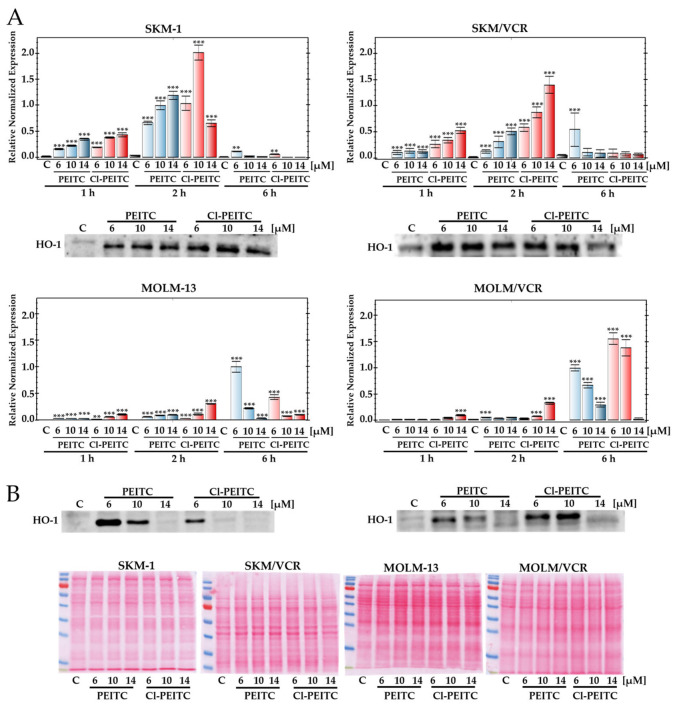
Effect of PEITC and Cl-PEITC on *HMOX1* expression on mRNA and protein levels. Human leukemia cell lines SKM-1, SKM/VCR, MOLM-13 and MOLM/VCR were treated with PEITC or Cl-PEITC (6, 10, and 14 µM). (**A**) For *HMOX1* mRNA expression analysis, RNA was isolated after 1, 2, and 6 h of treatment, while total protein extracts for (**B**) Western blotting analysis of HO-1 were collected after 6 h. Results are expressed as mean ± SD of three independent experiments. Statistical analysis was performed for qPCR data only using one-way ANOVA followed by Sidak’s post hoc test (** *p* < 0.01, *** *p* < 0.001 vs. control cells). Ponceau S staining was used as an internal loading control to verify the protein loading (60 µg/lane).

To determine the effect of activating the Nrf2 transcription factor on *HMOX1* expression, we measured both *HMOX1* mRNA (Figure 8A) and cytosolic HO-1 protein levels (Figure 8B). The tested compounds predominantly increased *HMOX1* expression at the transcript level. A marked difference was observed between the SKM-1/SKM-VCR and MOLM-13/MOLM-VCR cell variants. The former two sublines showed a significant increase in *HMOX1* transcript levels after 1 and 2 h of incubation with PEITC or Cl-PEITC (6, 10 and, 14 µM), followed by a decline after 6 h. In contrast, MOLM-13 and MOLM-VCR cells predominantly exhibited increased *HMOX1* transcript levels after 6 h of treatment with PEITC or Cl-PEITC at 6 µM. Higher concentrations of both ITCs reduced transcript levels relative to these increased values. *HMOX1* mRNA expression was therefore assessed at multiple early and later time points (1, 2, and 6 h) to capture the dynamic transcriptional response to oxidative stress, whereas HO-1 protein levels were determined at 6 h as a representative time point to capture early protein induction while allowing sufficient time for translation following transcriptional activation.

At the protein level, increased HO-1 expression was observed following treatment with 6 or 10 µM of either PEITC or Cl-PEITC. However, treatment with 14 µM resulted in a decrease relative to the induced levels.

### 2.8. DNA Fragmentation

**Figure 9:**
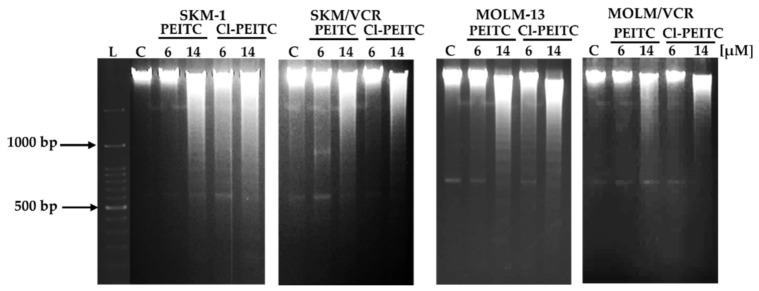
DNA fragmentation assay. Gel electrophoresis was performed to evaluate intranucleosomal DNA fragmentation in a 1.5% agarose gel. SKM-1, SKM/VCR, MOLM-13, and MOLM/VCR cell lines were treated with PEITC or Cl-PEITC at concentrations of 6 and 14 µM for 24 h. L: molecular weight marker; C: untreated control.

Agarose gel electrophoresis was performed to evaluate DNA fragmentation. Leukemia cells were incubated with the indicated concentrations of PEITC or Cl-PEITC (6 and 14 µM). DNA fragmentation was detected after 24 h of exposure to both PEITC and Cl-PEITC. Cl-PEITC was more effective, inducing detectable DNA fragmentation at 6 µM, whereas PEITC only induced fragmentation at 14 µM. DNA fragmentation was more pronounced in the parental cell lines than in the P-gp-positive sublines (Figure 9).

### 2.9. PEITC and Cl-PEITC Promoted Autophagy

To investigate the role of autophagy in the cellular response to PEITC and Cl-PEITC, we analyzed expression of the autophagy-related proteins LC3 and p62/SQSTM1. The conversion of LC3-I to LC3-II and changes in p62 levels are widely used indicators of autophagic activity.

**Figure 10:**
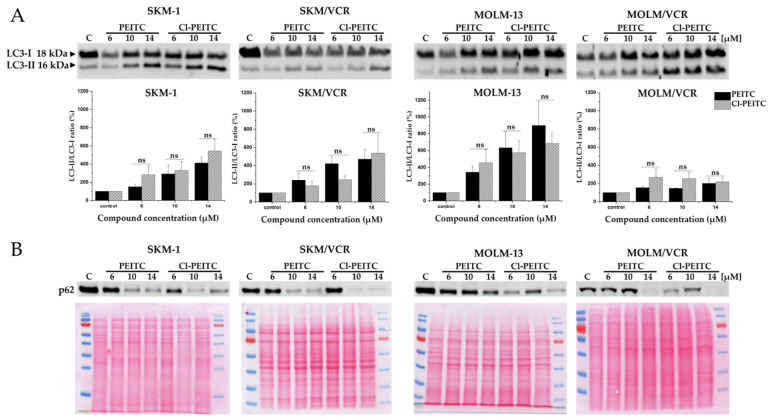
Effect of PEITC and Cl-PEITC on protein expression of autophagy-associated factors. Western blot analysis of (**A**) LC3-I and LC3-II and (**B**) p62 proteins was performed in SKM-1, SKM/VCR, MOLM-13, and MOLM/VCR cell lines following treatment with PEITC or Cl-PEITC (6, 10, and 14 µM) for 12 h. The LC3-II/LC3-I ratio was determined by densitometric analysis of immunoblots using ImageJ (NIH, version 1.52o). Data are presented as mean ± SD of three independent experiments. Statistical analysis was performed using two-way ANOVA followed by Tukey’s multiple comparisons test. No statistically significant (ns) differences were detected at the tested concentrations (PEITC vs. Cl-PEITC).

After treatment with PEITC and Cl-PEITC, the LC3-II/LC3-I ratio increased (Figure 10A), whereas p62 protein levels decreased (Figure 10B), consistent with enhanced autophagic activity in a concentration-dependent manner. No significant interaction between compound type and concentration was detected across the tested cell lines, indicating that both compounds produced comparable concentration-dependent effects on LC3 conversion. Accordingly, LC3 conversion was influenced primarily by concentration rather than by the specific ITC used. Notably, MOLM/VCR cells showed only minor changes in LC3 conversion across all treatment conditions, indicating a less pronounced autophagic response than that observed in the other leukemia cell lines.

## 3. Discussion

Various small molecules produced by plant metabolism represent a rich source of medicinal agents and bioactive compounds. These substances are widely used to treat numerous diseases, including cancer [35]. More than 60% of currently used anticancer drugs are derived directly or indirectly from natural sources [36].

Glucosinolates, which are converted into ITCs through myrosinase-catalyzed hydrolysis, are naturally present in various plant species, particularly those belonging to the Brassicaceae family. ITCs are important bioactive compounds that regulate antioxidant processes and have attracted attention due to their potential therapeutic applications [37]. In addition, they exhibit anticancer, antimicrobial, and anti-inflammatory properties [38,39]. PEITC is a compound consisting of a benzene ring linked to an ethyl chain and an ITC functional group. It has attracted considerable interest due to its diverse biological activities, including anticancer properties [40]. PEITC has been shown to inhibit cell proliferation, interfere with cell cycle progression, suppress DNA replication, and induce DNA damage [41]. These effects may contribute to its selective activity against cancer cells, which frequently exhibit altered DNA damage response and repair mechanisms. Moreover, PEITC has demonstrated considerable potential for cancer prevention and therapy by modulating multiple molecular pathways involved in tumor initiation and progression. It affects key cellular processes, including proliferation, migration, and metastasis, primarily by inducing apoptosis and cell cycle arrest [42]. Consistent with these mechanisms, PEITC has exhibited anticancer activity across a broad spectrum of malignancies, including leukemia [43].

To further enhance the biological activity of naturally occurring ITCs, structural analogs of these compounds have been investigated [44]. The introduction of halogen substituents into the aromatic structures of natural ITCs has been reported to improve their physicochemical properties, including increased lipophilicity and enhanced ability to penetrate cellular targets. Additionally, the presence of halogen substituents in aromatic compounds has been associated with enhanced cytostatic, antiproliferative, and antimigratory effects in tumor cells [15,17].

For example, the higher cytotoxic activity of 4-chloro-β-phenylisothiocyanate compared to β-phenylisothiocyanate was first reported in 1968 [14]. However, despite these observations, information regarding the effects of halogenated derivatives of aromatic ITCs on leukemic cells remains limited. Therefore, the aim of the present study was to determine whether Cl-PEITC exerts stronger antileukemic activity than PEITC in acute myeloid leukemia cells and their P-gp-positive drug-resistant variants. Compared with PEITC, chlorination of the benzene moiety in Cl-PEITC may alter its physicochemical properties, including lipophilicity and interactions with biological targets. These modifications may affect intracellular behavior and biological activity, thereby partially contributing to the enhanced efficacy of Cl-PEITC observed in our experiments [19,45].

Using two human leukemia cell lines (SKM-1 and MOLM-13) and their drug-resistant variants (SKM/VCR and MOLM/VCR), we demonstrated that PEITC and Cl-PEITC inhibit cell proliferation and reduce metabolic activity in a concentration- and time-dependent manner, as measured by the MTS assay (Figure 2 and Figure 3). Consistent with our hypothesis, Cl-PEITC showed stronger cytotoxicity across all examined cell lines, including P-gp-positive SKM/VCR and MOLM/VCR cells.

We confirmed significant effects of both compound type and concentration, as well as a significant interaction between them, indicating that the biological response depended on both the applied concentration and the chemical structure of the tested ITC. Interestingly, the differences between PEITC and Cl-PEITC were most pronounced at intermediate concentrations, whereas at higher concentrations both compounds produced comparable maximal cytotoxic effects. This observation suggests that chlorination primarily enhances the potency rather than the maximal efficacy of PEITC, shifting its biological activity toward lower concentrations and enabling similar levels of growth inhibition at reduced doses.

Unlike PEITC, which showed lower efficacy in P-gp-positive cell lines than in their drug-sensitive counterparts, Cl-PEITC exhibited comparable cytotoxicity in drug-sensitive and drug-resistant cells (Table 1). This finding suggests that Cl-PEITC retains its antileukemic activity in P-gp positive cells and reduces the impact of P-gp associated MDR. This effect may be directly or indirectly related to P-gp efflux activity. P-gp overexpression is accompanied by multiple molecular alterations that contribute to reduced sensitivity to anticancer compounds, including those that are not direct substrates of this transporter, in addition to drug efflux [46]. The ability of Cl-PEITC to achieve comparable biological responses at lower concentrations may therefore be an advantageous feature for further investigation in the context of multidrug-resistant leukemia.

Our data further indicate that Cl-PEITC induced apoptosis in a higher proportion of cells in both P-gp-negative, drug-sensitive cells and their P-gp-positive multidrug-resistant variants (Figure 4). This increased apoptotic response correlated with activation of the caspase cascade and PARP1 cleavage (Figure 5). Full-length PARP1, a 116 kDa protein, is involved in DNA repair and chromatin organization. During apoptosis, caspases, particularly caspase-3, cleave PARP1 into an 89 kDa fragment, thereby inactivating its function [47].

Activation of initiator caspases (caspases-8 and -9) and the executioner caspase-3 indicates that PEITC and, more notably, Cl-PEITC activate both the extrinsic and intrinsic apoptotic pathways. This ultimately leads to the characteristic fragmentation of nuclear DNA observed during apoptosis (Figure 9). These findings are consistent with previous reports on PEITC-induced apoptosis [48,49] and suggest that the chlorination of the aromatic ring in Cl-PEITC enhances its ability to trigger multiple apoptotic signaling pathways.

The observed differences between PEITC and Cl-PEITC indicate that the apoptotic response did not increase uniformly with concentration for either compound. Instead, the difference between PEITC and Cl-PEITC became more pronounced, suggesting that chlorination modifies the concentration–response relationship rather than simply increasing overall potency. This observation points to compound-specific effects on the regulation of apoptotic signaling.

Increased ROS production, particularly within the mitochondrial compartment, plays a key role in inducing apoptosis, primarily via the intrinsic pathway [50]. Excessive intracellular ROS accumulation is a hallmark of oxidative stress. Maintaining ROS homeostasis is essential for cellular function because low-to-moderate ROS levels regulate signaling pathways involved in differentiation, proliferation, and survival. However, elevated ROS levels can damage DNA, proteins, and lipids, ultimately leading to cell death [51,52]. Therefore, modulation of intracellular ROS levels or impairment of antioxidant defense systems may represent an effective strategy to enhance therapeutic responses and overcome drug resistance [53,54].

The pattern of ROS induction differed between the leukemia models (Figure 6). While SKM-derived cells showed predominantly additive effects of concentration and compound type, MOLM-derived cells showed a significant interaction between these factors, indicating that the difference between PEITC and Cl-PEITC became more pronounced at specific concentrations. These findings suggest that the effect of chlorination on redox regulation may be cell type-dependent and influenced by differences in antioxidant capacity, glutathione metabolism, or stress-response signaling pathways. The observed increase in ROS is consistent with the proposed mechanism of action of isothiocyanates, in which disruption of cellular redox homeostasis contributes to the activation of apoptotic signaling [55]. It should be noted that DCFH-DA is a non-specific indicator of intracellular oxidative stress; therefore, the present data do not allow identification of the specific reactive oxygen species responsible for the observed increase in fluorescence. Although ROS accumulation correlated with apoptotic markers and autophagy-related changes, the present data do not allow determination of whether ROS generation is the primary driver of cell death or a downstream consequence of cellular stress.

Another manifestation of the altered redox balance after treatment with the investigated ITCs was the modulation of *HMOX1* gene expression (Figure 8). *HMOX1* encodes heme oxygenase-1 (HO-1), an enzyme that catalyzes the degradation of heme into carbon monoxide, biliverdin, and free Fe^2+^ ions [56]. *HMOX1* expression increased in a concentration- and time-dependent manner following PEITC and Cl-PEITC treatment during the first 1–2 h of incubation, consistent with an early adaptive response to oxidative stress. However, *HMOX1* expression decreased after 6 h of incubation with both ITCs.

Consistent with this decline, apoptosis became evident after 6 h and 12 h of treatment (Supplementary Data Figure S2 and Figure 4, respectively). These findings suggest that the initial cellular stress response, activated under pro-oxidative conditions, was insufficient to maintain cellular adaptation. This was accompanied by the emergence of programmed cell death. The relationship between *HMOX1* expression and apoptosis is complex and context-dependent. However, HO-1 is often associated with cytoprotective and anti-apoptotic functions. Consequently, knocking down HO-1 has been reported to promote apoptosis and enhance the cytotoxic effects of doxorubicin in cancer cells [57].

The cellular response to increased oxidative stress following PEITC and, more prominently, Cl-PEITC at lower concentrations was reflected in the activation of the NRF2–KEAP1 signaling axis, as evidenced by increased NRF2 protein levels and reduced KEAP1 expression (Figure 7). Consistent with our findings, Ko et al. (2025) reported increased NRF2 expression following PEITC treatment [58]. These authors also demonstrated that PEITC can modify the critical thiol group of the KEAP1cysteine 151 residue, thereby affecting its regulatory function. Therefore, in addition to changes in protein expression, functional alterations in KEAP1 should be considered.

At the highest concentrations of Cl-PEITC and, to a lesser extent, PEITC, levels of the NRF2 protein decreased, which is consistent with the previously described induction of apoptosis. Treatment with ITCs also increased levels of the 16 kDa form of LC3, suggesting activation of autophagy (Figure 10).

A growing body of evidence indicates that ROS are involved in the regulation of autophagy, which may function as a cytoprotective mechanism or, under conditions of excessive cellular stress, contribute to apoptotic cell death [59]. The interplay between the autophagy and apoptosis pathways may therefore contribute to the anticancer effects of PEITC, consistent with previous reports [60,61].

At the same time, levels of the p65 member of the canonical NF-κB pathway decreased with increasing concentrations of both compounds. These findings are consistent with known crosstalk between NRF2 and NF-κB signaling pathways and with previous reports demonstrating that ITCs suppress NF-κB signaling, at least in part, by inhibiting IKK activity. This prevents IκB degradation and subsequent NF-κB activation [62]. Notably, changes in both NRF2 and NF-κB expression were more pronounced in P-gp-positive resistant variants. This suggests that redox modulation and stress-response pathways may be differentially regulated in drug-resistant cells.

Autophagy is a tightly regulated lysosome-dependent degradation pathway that maintains cellular homeostasis by removing damaged organelles and protein aggregates. A central event in autophagosome formation is the conversion of cytosolic LC3-I to its lipidated form LC3-II through conjugation with phosphatidylethanolamine. LC3-II is incorporated into autophagosomal membranes and participates in cargo sequestration and autophagosome maturation. Following fusion with lysosomes, both LC3-II and the engulfed cargo are degraded. The adaptor protein p62/SQSTM1 mediates the selective recruitment of ubiquitinated proteins into autophagosomes and is itself degraded during autophagic flux. Therefore, an increased LC3-II/LC3-I ratio accompanied by decreased p62 levels is commonly used as an indicator of active autophagic degradation and is widely applied to assess ITC-induced autophagy in leukemia models [63].

It has been proposed that the same stimuli can trigger both autophagy and apoptosis, both of which are regulated by complex, interconnected signaling pathways [64]. Beyond its degradative function, autophagy is a dynamic, stress-adaptive response whose outcome depends on the intensity and duration of cellular stress. In cancer, it plays a dual role, acting either as a tumor-suppressive mechanism by limiting the accumulation of cellular damage or as a pro-survival pathway that supports metabolic adaptation and the maintenance of neoplastic cells under adverse conditions [65]. However, it can also support tumor development by maintaining the metabolism, growth potential, and cellular homeostasis of neoplastic cells [66]. Consistent with this context-dependent function, we previously showed that aliphatic ITCs such as AITC and SFN predominantly induce autophagy in leukemia cells, whereas apoptosis remains a secondary response [7,8].

In this study, PEITC and Cl-PEITC increased LC3 conversion (Figure 10A) and decreased p62 protein levels (Figure 10B) across most leukemia cell lines, indicating activation of autophagic flux. This response was primarily concentration-dependent, with only minor differences between compounds. No significant interaction between compound type and concentration was observed across all tested cell lines, suggesting that both ITCs regulate autophagy through comparable dose-dependent mechanisms. At the highest Cl-PEITC concentration, particularly in MOLM-13 cells, reduced autophagic activity may reflect impaired autophagic flux or a shift toward apoptosis under conditions of excessive stress. Increased ROS production may be associated with mitochondrial and lysosomal stress, which could influence autophagic flux rather than autophagy initiation. Under these conditions, increased cellular stress may be associated with a shift from adaptive autophagic responses toward apoptotic or necrotic cell death pathways. Overall, these findings support a dose-dependent hormetic response, in which mild stress activates protective autophagy, whereas excessive stress overwhelms cellular homeostasis and promotes cell death rather than survival.

## 4. Materials and Methods

### 4.1. Chemicals

PEITC (Phenethyl isothiocyanate, product code: 253731) and Cl-PEITC (2-(4-Chlorophenethyl)isothiocyanate, product code: 591378) (Figure 1) were purchased from Sigma-Aldrich (Merck Life Science, Bratislava, Slovakia).

### 4.2. Methods

#### 4.2.1. Cell Culture

SKM-1 (ACC 547) is a human acute myeloid leukemia cell line derived from the peripheral blood of a 76-year-old male with acute myeloid leukemia (AML M5) that progressed to myelodysplastic syndrome (MDS). MOLM-13 (ACC 554) is a cell line established from the peripheral blood of a 20-year-old male with acute myeloid leukemia AML FAB M5a at relapse in 1995 after initial MDS. Both cell cultures were obtained from Leibniz-Institut-Deutsche Sammlung von Mikroorganismen und Zellkulturen GmbH (Braunschweig, Germany). The drug-resistant cell lines SKM/VCR and MOLM/VCR with overexpression of the drug transporter *ABCB1* were established by long-term cultivation with the anticancer drug vincristine (VCR; Sigma-Aldrich, St. Louis, MO, USA), whose concentrations were gradually increased to a final concentration of 60 nM, as described previously [67]. The cells were cultured in RPMI 1640 medium with L-glutamine (Sigma-Aldrich, St. Louis, MO, USA) supplemented with 12% heat-inactivated fetal bovine serum (FBS; Cytiva HyClone, Bad Kreuznach, Germany), penicillin (100 U/mL), and streptomycin (50 mg/L) (both purchased from Sigma-Aldrich, St. Louis, MO, USA) in a humidified atmosphere with 5% CO_2_ at 37 °C. Cells were subcultured three times a week. All cell lines used in this study were routinely screened for mycoplasma contamination and tested negative throughout the experimental period.

#### 4.2.2. Viable Cells Counting

The effects of PEITC or Cl-PEITC on cell proliferation were determined by counting the absolute number of viable cells by measuring plasma membrane integrity using a CASY Model TT Cell Counter (Roche Applied Sciences, Madison, WI, USA) according to the manufacturer’s protocol. Briefly, SKM-1, SKM/VCR, MOLM-13, and MOLM/VCR cells (3 × 10^5^ cells/mL) were seeded into 24-well plates (2 mL per well) and treated with PEITC (4–24 µM) or Cl-PEITC (4–40 µM) for 24 h and 48 h. The results represent the mean ± SD of at least three independent experiments. We determined the drug concentration that inhibited cell proliferation to 50% of the control (IC_50_); these values were measured from three independent triplicate experiments for each treatment. Nonlinear regression analysis was performed using an equation previously published [11].

#### 4.2.3. Cell Viability Assay and Determination of IC_50_ Values

The cytotoxic effects of PEITC or Cl-PEITC were assessed by measuring cellular metabolic activity using 3-(4,5-dimethylthiazol-2-yl)-5-(3-carboxymethoxyphenyl)-2-(4-sulfophenyl)-2H-tetrazolium inner salt (MTS assay) according to the manufacturer’s protocol (Promega, Madison, WI, USA). This allowed us to determine the IC_50_ concentrations of the studied agents. The tested compounds were dissolved in dimethyl sulfoxide (DMSO; CentralChem, Bratislava, Slovakia) and diluted with RPMI 1640 culture medium. SKM-1, SKM/VCR, MOLM-13, and MOLM/VCR cells were seeded at a density of 6 × 10^5^ cells/well in 24-well plates and treated with PEITC (4–24 µM) or Cl-PEITC (4–40 µM) for 24 h and 48 h at 37 °C. After incubation, 100 µL of each sample was added to a 96-well plate containing 20 µL of MTS assay solution, and the plate was incubated for 3 h. The absorbance of each well was read at 490 nm using an Epoch 2 microplate reader (BioTek Instruments, Inc., Winooski, VT, USA). Each experiment was performed in six replicates and repeated at least three times. The average relative absorbance was used as a measure of the cells’ MTS reductase activity. The drug concentrations that inhibited cell proliferation by 50% of the control (IC_50_) were determined as previously described [11]. The resistance index (RI) was calculated as the ratio of the IC_50_ of the resistant cells to the IC_50_ of the parental cells.

#### 4.2.4. Flow Cytometry Analysis of Cell Death

Apoptosis induction was determined using the Annexin V-FLUOS Staining Kit (Roche Diagnostics, Mannheim, Germany) according to the manufacturer’s instructions. Briefly, SKM-1, SKM/VCR, MOLM-13, and MOLM/VCR cells (1.25 × 10^6^ cells/well) were seeded in six-well plates and grown overnight. After 24 h, the cells were treated with PEITC or Cl-PEITC at concentrations of 6, 10, and 14 µM for 3, 6, or 12 h. After treatment, the cells (1 × 10^6^ cells) were harvested, washed with PBS, and resuspended in 100 µL of annexin V-FLUOS labeling solution/propidium iodide (PI). The cells were stained in the dark for 15 min at room temperature. Annexin V-FITC binding and PI staining were analyzed using an Accuri C6 flow cytometer (BD Bioscience, San Jose, CA, USA). Both early apoptotic cells (Annexin V-positive and PI-negative) and late apoptotic cells (Annexin V-positive and PI-positive) were included in the cell death determinations. For each analysis, 10,000 events were recorded.

#### 4.2.5. Western Blotting

SKM-1, SKM/VCR, MOLM-13, and MOLM/VCR cells (2.5 × 10^5^ cells/mL) were seeded in cell culture flasks and grown overnight. After 24 h, the cells were treated with 6, 10, or 14 µM of PEITC or Cl-PEITC and incubated for 6 h or 12 h. The treated cells were harvested and lysed with RIPA lysis buffer containing a protease inhibitor cocktail (Sigma-Aldrich, St. Louis, MO, USA) to extract the total proteins. The samples were heated at 100 °C for 15 min. Then, the protein samples (60 µg) were separated by SDS-PAGE gel electrophoresis according to Laemmli’s published protocol [68] and transferred to a nitrocellulose membrane (GE Healthcare Europe GmbH, Vienna, Austria). Nonspecific proteins were blocked with 5% nonfat dry milk in TBS containing 0.1% Tween-20 (TTBS) for 2 h. The membrane was then incubated with a specific primary antibody against each protein: Heme Oxygenase 1 (A-3),sc-136960; Keap1 (G-2), sc-365626; NFκB p65 (F-6), sc-8008; and Nrf2 Antibody (A-10), sc-365949, all from Santa Cruz Biotechnology, Dallas, TX, USA. Anti-Caspase 3 Active (C8487), LC3B antibody (L7543), and Anti-Actin N-terminal (A2103) were all from Sigma-Aldrich, St. Louis, MO, USA. Anti-Cleaved PARP1 antibody [Y34] (ab32561) was from Abcam, Cambridge, UK. Anti-Caspase 8 (cleaved Asp384) Antibody (A95120) and Anti-Caspase 9 (cleaved Asp330) Antibody (A94477) were both from Antibodies.com, Cambridge, UK. p62 Antibody (PA5-27247) was from Invitrogen, Thermo Fisher Scientific, Waltham, MA, USA. Anti-GAPDH (MAB374) was from EMD Millipore Chemicals, Billerica, MA, USA. Incubation was conducted overnight at 4 °C followed by supplementation of the appropriate horseradish peroxidase- (HRP-)-conjugated secondary antibodies IgG kappa binding protein (m-IgGκ BP) or mouse anti-rabbit IgG-HRP (both purchased from Santa Cruz Biotechnology, Dallas, TX, USA) for 2 h at room temperature. The secondary antibodies were diluted with 2.5% skim milk in TTBS. HRP signals were visualized using an ECL detection system (GE Healthcare Europe GmbH, Vienna, Austria) on an Amersham Imager 600 system (GE Healthcare Europe GmbH, Pittsburgh, PA, USA). Transfer efficiency and equal protein loading were assessed by staining the membranes with Ponceau S staining solution (Thermo Fisher Scientific, Waltham, MA, USA) for 10 min at room temperature with gentle agitation, serving as an internal control. The membranes were scanned, then rinsed with distilled water for 2–3 min until the stain was completely removed. The membranes were then blocked and incubated with the appropriate primary and secondary antibodies. The intensity of the protein bands was quantified using the National Institutes of Health’s ImageJ 1.52o program. Ponceau S total protein staining was used for quantitative total-protein normalization across samples and served to confirm equal protein loading and transfer efficiency in all Western blot analyses.

#### 4.2.6. Measurement of Intracellular ROS Production

Intracellular ROS levels were measured in SKM-1, SKM/VCR, MOLM-13 and MOLM/VCR cell lines using 2′,7′-dichlorodihydrofluorescein diacetate (DCFH-DA, Sigma-Aldrich, St. Louis, MO, USA), an oxidation-sensitive fluorescent dye. This assay was used to evaluate ROS levels induced by treatment with PEITC or Cl-PEITC. DCFH-DA passively diffuses across cell membranes and is subsequently deacetylated by intracellular esterases to yield 2′,7′-dichlorodihydrofluorescein (DCFH_2_). DCFH_2_ is a non-fluorescent compound. DCFH_2_ is then oxidized by ROS to form the fluorescent product, 2′,7′-dichlorofluorescein (DCF) [69]. Cells were seeded at 1.25 × 10^6^ cells/well in 6-well plates and incubated for 24 h, followed by treatment with 6, 10 or 14 µM PEITC or Cl-PEITC for 20 min. Hydrogen peroxide (0.1%, 5 min) was used as a positive control for ROS induction. Subsequently, 1 × 10^6^ cells were harvested, washed with FBS-free RPMI 1640 medium, and resuspended in 300 µL FBS-free RPMI 1640 medium containing 5 µM DCFH-DA. Samples were incubated for 30 min at 37 °C in an atmosphere supplemented by 5% CO_2_ and protected from light, washed with PBS, and resuspended in 300 µL PBS. Fluorescence intensity was measured using flow cytometry (Accuri C6, BD Biosciences, San Jose, CA, USA) with the FL1 (FITC) detector. The median fluorescence intensity was determined by counting 10,000 events from the gated cell population, and all experiments were performed in triplicate.

#### 4.2.7. DNA Fragmentation Analysis

SKM-1, SKM/VCR, MOLM-13, and MOLM/VCR cells were seeded at a density of 3 × 10^5^ cells/mL in 20 mL Petri dishes and treated with 6 or 14 µM PEITC or Cl-PEITC for 24 h. Subsequently, 4 × 10^6^ cells were harvested and genomic DNA was isolated using the AllPrep DNA/RNA Mini Kit (Qiagen, Hilden, Germany), according to the manufacturer’s instructions, to assess nuclear DNA fragmentation. DNA fragmentation was analyzed by electrophoresis on a 1.5% agarose gel containing 0.5 µg/mL ethidium bromide (Sigma-Aldrich, St. Louis, MO, USA). A DNA Ladder High 100 bp (highQu GmbH, Kraichtal, Germany) was used as a molecular weight marker. The gels were visualized and documented using an Amersham Imager 600 system (GE Healthcare Europe GmbH, Pittsburgh, PA, USA).

#### 4.2.8. RNA Isolation and RT-qPCR Analysis

The SKM-1, SKM/VCR, MOLM-13, and MOLM/VCR cell lines were seeded at a density of 1.25 × 10^6^ cells per well in six-well plates and incubated for 24 h. The cells were then treated with PEITC or Cl-PEITC at various concentrations (6, 10, and 14 µM) for 1, 2, or 6 h. Total RNA was isolated from the cells using TRIREAGENT^®^ (MRC, Cincinnati, OH, USA) according to the manufacturer’s protocol. The concentration and purity of the RNA were measured by a NanoPhotometer^®^ (Implen GmbH, Munich, Germany) using the A260/A280 ratio. Reverse transcription (RT) was performed with 1 µg of total RNA using a RevertAid™ First Strand cDNA Synthesis Kit (Thermo Fisher Scientific, Waltham, MA, MA) according to the manufacturer’s instructions. Relative *HMOX1* mRNA expression levels were quantified by qPCR. The sequences of the forward and reverse primers used for housekeeping gene *RNA18SN1* and *HMOX1* are as follows: *RNA18SN1* forward: 5’-TGAAACTGCGAATGGCTCA-3’; *RNA18SN1* reverse: 5’-CCGTCGGCATGTATTAGCTC-3’; *HMOX1* forward: 5’-CAACAAAGTGCAAGATTCTG-3’; *HMOX1* reverse: 5’-TGCATTCACATGGCATAAAG-3’. These primers were synthesized by Merck (Merck Life Science, Bratislava, Slovakia). The data were normalized to *RNA18SN1* and analyzed using the 2^−ΔΔCt^ method. The data was analyzed with Bio-Rad CFX Manager 3.1 software.

## Figures and Tables

**Table 1:** IC_50_ values for PEITC and Cl-PEITC determined by CASY TT cell counting and MTS assay in parental and MDR cell lines after 24 and 48 h.

The Cells Were Incubated for 24 h				
	CASY TT Cell Counting		MTS Viability Estimation	
Cell Line	PEITC IC_50_	Cl-PEITC IC_50_	PEITC IC_50_	Cl-PEITC IC_50_
	(µM)	(µM)	(µM)	(µM)
SKM-1	6.459 ± 0.834	4.683 ± 0.404	8.710 ± 0.272	5.230 ± 0.258
SKM/VCR	8.357 ± 0.588	4.698 ± 0.236	10.630 ± 0.244	5.692 ± 0.276
MOLM-13	11.250 ± 1.244	6.973 ± 0.858	14.850 ± 0.308	8.641 ± 0.334
MOLM/VCR	18.630 ± 1.772	8.462 ± 0.686	23.030 ± 0.620	9.992 ± 0.324
**The cells were incubated for 48 h**				
SKM-1	6.769 ± 1.046	5.456 ± 0.324	9.157 ± 0.370	6.209 ± 0.240
SKM/VCR	8.761 ± 0.442	5.032 ± 0.290	10.440 ± 0.290	6.031 ± 0.208
MOLM-13	11.510 ± 1.036	8.147 ± 0.732	14.720 ± 0.266	8.720 ± 0.346
MOLM/VCR	20.040 ± 1.674	9.758 ± 0.664	23.570 ± 0.616	11.610 ± 0.342

Median-inhibitory concentration (IC_50_) values for PEITC or Cl-PEITC were calculated by nonlinear regression of cell proliferation data according to the equation [11].

**Table 2:** Comparison of resistance index (RI) values for PEITC and Cl-PEITC obtained by CASY cell counting and MTS assay.

		Resistance Index (RI)			
Cell Variant	Time	Cell Counting		MTS	
	(h)	PEITC	Cl-PEITC	PEITC	Cl-PEITC
SKM/VCR vs. SKM-1	24	1.29	1.00	1.22	1.09
MOLM/VCR vs. MOLM-13	24	1.66	1.21	1.55	1.16
SKM/VCR vs. SKM-1	48	1.29	0.92	1.14	0.97
MOLM/VCR vs. MOLM-13	48	1.74	1.20	1.60	1.33

RI—calculated as the ratio of the IC_50_ of the resistant cells to the IC_50_ of the sensitive counterparts.

## Data Availability

The original contributions presented in this study are included in the article/Supplementary Material. Further inquiries can be directed to the corresponding authors.

## Supplementary Materials

The following supporting information can be downloaded at https://www.mdpi.com/article/10.3390/ijms27135869/s1.

## Abbreviations

µM	Micro molar
AITC	Allyl isothiocyanate
AML	Acute myeloid leukemia
ANOVA	Analysis of variance
BITC	Benzyl isothiocyanate
Cl-PEITC	2-(4-Chlorophenethyl) isothiocyanate
DCF	dichlorofluorescein
DCFH_2_	2′,7′-dichlorodihydrofluorescein
DCFH-DA	2′,7′-dichlorodihydrofluorescein diacetate
DMSO	Dimethyl sulfoxide
FACS	Fluorescence-Activated Cell Sorting
FBS	Fetal bovine serum
FDA	U.S. Food and Drug Administration
Fe^2+^	Ferrous iron
GAPDH	Glyceraldehyde-3-phosphate dehydrogenase
HMOX1	Heme oxygenase 1 gene
HO-1	Heme oxygenase 1
HRP	horseradish peroxidase
IC_50_	Half-maximal inhibitory concentration
IKK	IκB kinase complex
ITC	Isothiocyanate
KEAP1	Kelch-like ECH-associated protein 1
LC3	Microtubule-associated protein 1A/1B-light chain 3
MDR	Multidrug resistance
MDS	Myelodysplastic syndrome
MTS	3-(4,5-dimethylthiazol-2-yl)-5-(3-carboxymethoxyphenyl)-2-(4-sulfophenyl)-2H-tetrazolium inner salt
MTT	3-(4,5-dimethylthiazol-2-yl)-2,5-diphenyltetrazolium bromide.
NAD(P)H	Nicotinamide adenine dinucleotide phosphate
NF-κB	Nuclear factor kappa-light-chain-enhancer of activated B cells
NRF2	Nuclear factor erythroid 2-related factor 2
PARP	Poly(ADP-ribose) polymerase
PBS	Phosphate-buffered saline
PEITC	Phenethyl isothiocyanate
P-gp	P-glycoprotein
PI	Propidium iodide
qPCR	Quantitative polymerase chain reaction
RelA	NF-κB p65
RI	Resistance index
ROS	Reactive oxygen species
RT	Reverse transcription
SD	Standard deviation
SFN	Sulforaphane
TBS	Tris-Buffered Saline
TTBS	Tris-Buffered Saline with Tween 20
VCR	Vincristine
