# Supplementary material for: Chlorination of Phenethyl Isothiocyanate Potentiates Cytotoxicity and Apoptosis in Multidrug-Resistant Leukemia Cells
Source: Int J Mol Sci. 2026 Jun 29;27(13):5869. doi: 10.3390/ijms27135869 (PMC13362159; doi:10.3390/ijms27135869)
Supplement: Supplementary file 1 [file ijms-27-05869-s001.zip › ijms-4368470-supplementary.pdf]

International Journal of Molecular Sciences

MDPI

Article

Chlorination of Phenethyl Isothiocyanate Potentiates Cytotoxicity and Apoptosis in Multidrug-Resistant Leukemia Cells

Alberto Yoldi Vergara [1,†], Anna Bertova [1,†], Szilvia Kontar [1], Martina Ksinanova [1], Kristina Simonicova [1], Martin Simkovic [2], Zdena Sulova [1], Albert Breier [1,2*] and Denisa Imrichova [1,2*]

[1] Institute of Molecular Physiology and Genetics, Centre of Biosciences, Slovak Academy of Sciences, Dúbravská Cesta 9, 841 04 Bratislava, Slovakia
[2] Institute of Biochemistry and Microbiology, Faculty of Chemical and Food Technology, Slovak University of Technology in Bratislava, Radlinského 9, 812 37 Bratislava, Slovakia
* Correspondence, albert.breier@stuba.sk (A.Br.); denisa.imrichova@savba.sk (D.I.)
† These authors contributed equally to this work.

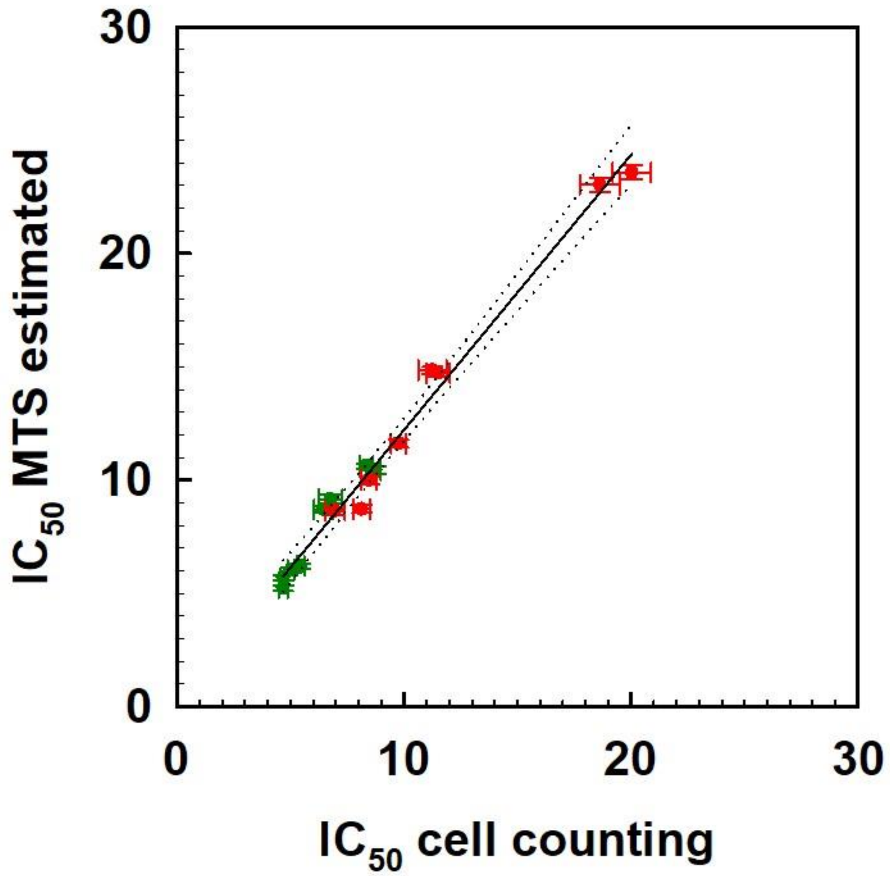

Figure S1. Correlation between IC_{50} values obtained by direct cell counting on the Casy TT cell counter and IC_{50} values obtained using the MTS assay. The IC_{50} values obtained by direct cell counting for SKM-1 and SKM/VCR (red symbols) and MOLM-13 and MOL/VCR (green symbols), which were incubated for 24 and 48 h in the presence of PEITC or Cl-PEITC, were plotted against the corresponding IC_{50} values obtained by the MTS assay. Linear regression, the correlation coefficient, and the 99% confidence interval were calculated using SigmaPlot 2002 for Windows, version 8.02.

International Journal of
Molecular Sciences

MDPI

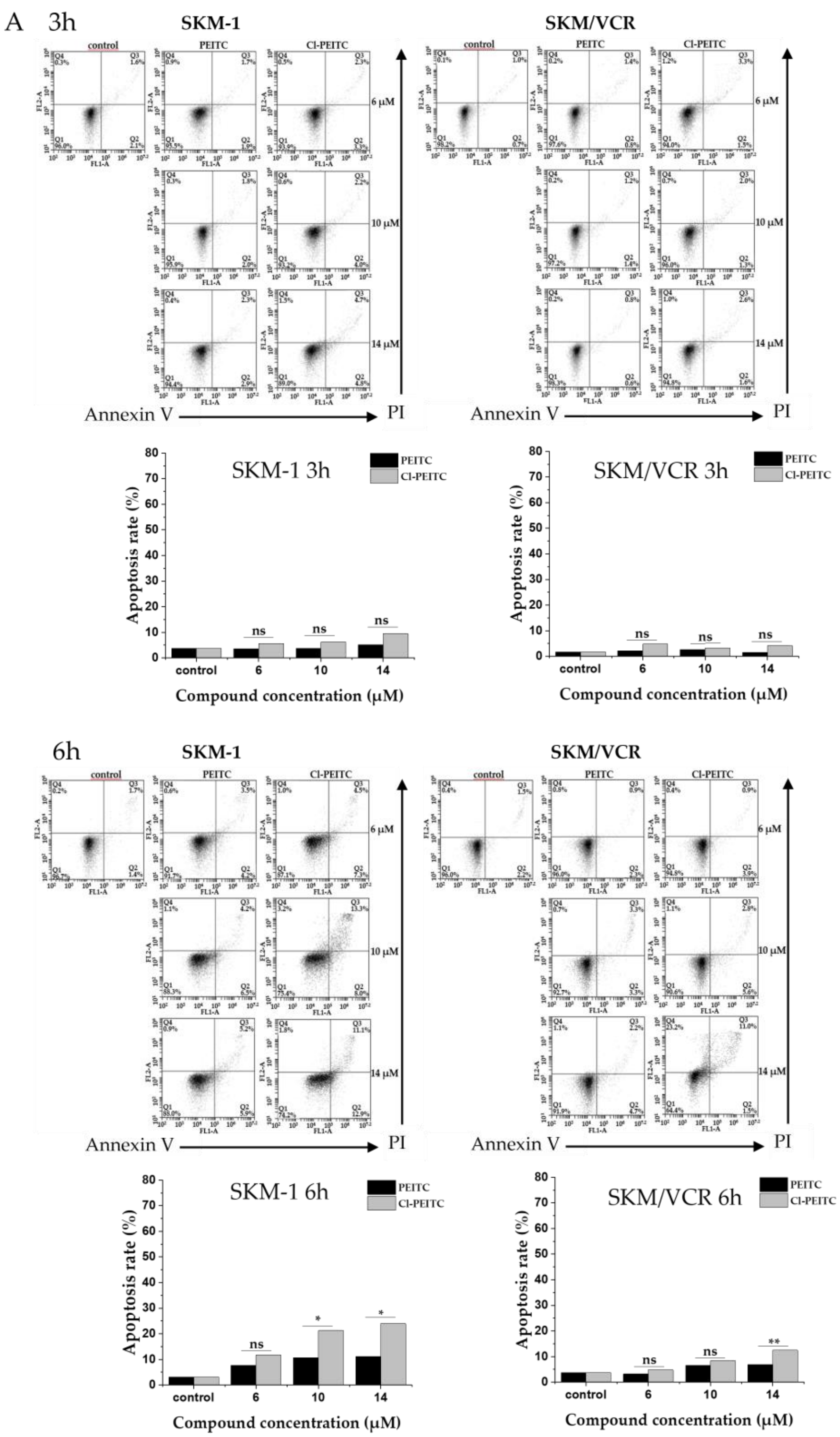

International Journal of *Molecular Sciences*

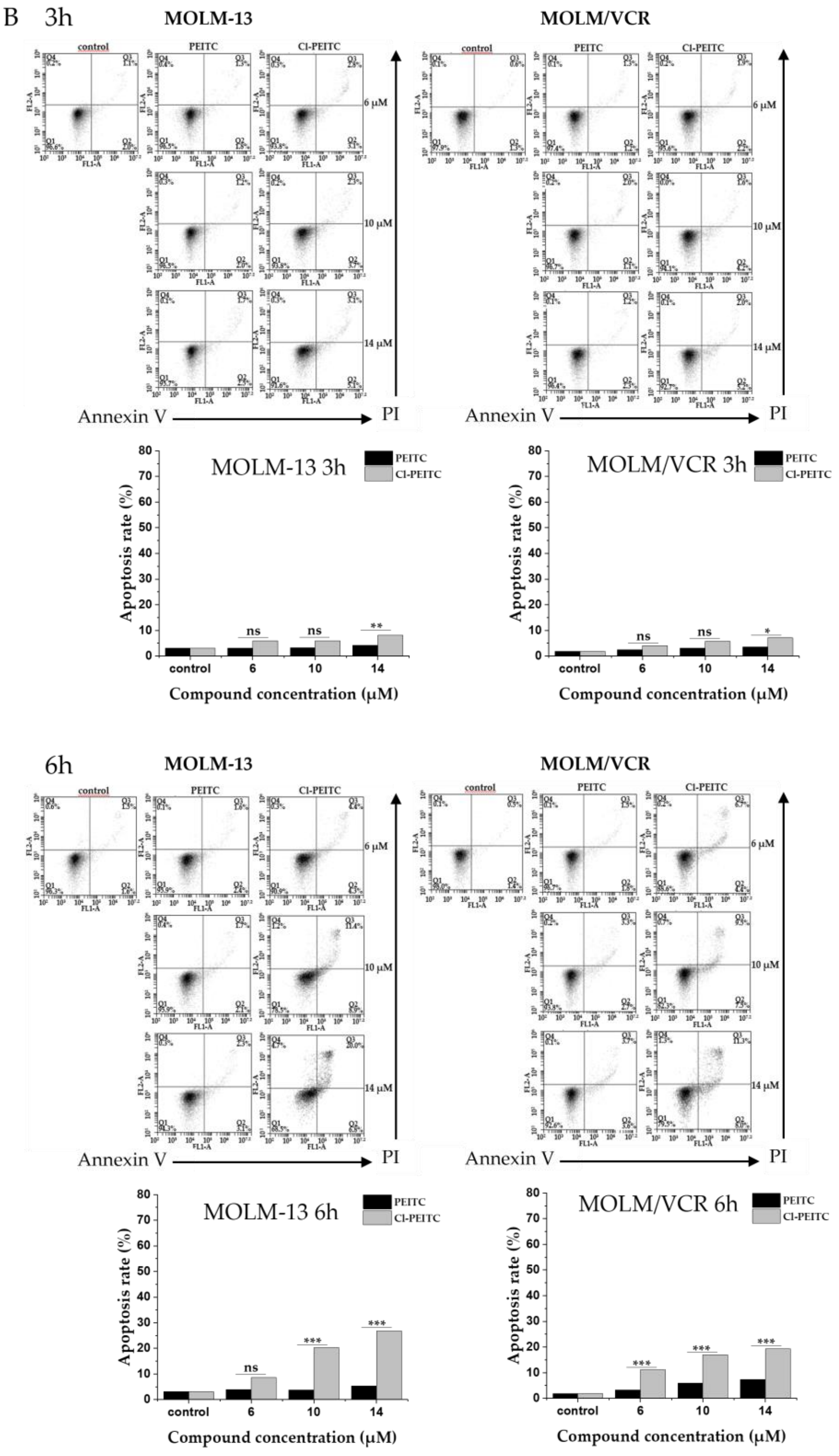

Figure S2. Apoptosis induced by PEITC and Cl-PEITC in (A) SKM-1, SKM/VCR, (B) MOLM-13, and MOLM/VCR cells. The cells were treated with different concentrations of PEITC or Cl-PEITC (6, 10, and 14 μM) for 3 h and 6 h. Apoptosis induction was detected using an Annexin V-FITC kit and flow cytometry as described in Materials and Methods. Each figure includes a representative cytogram and a quantitative data presentation. Statistical significance was assessed using two-way ANOVA with Tukey's post hoc multiple-comparison test, allowing pairwise evaluation of treatment concentration and compound type (PEITC vs. Cl-PEITC). Statistical significance was defined as * $p < 0.05$, ** $p < 0.01$, and *** $p < 0.001$; ns – not significant.

International Journal of
Molecular Sciences

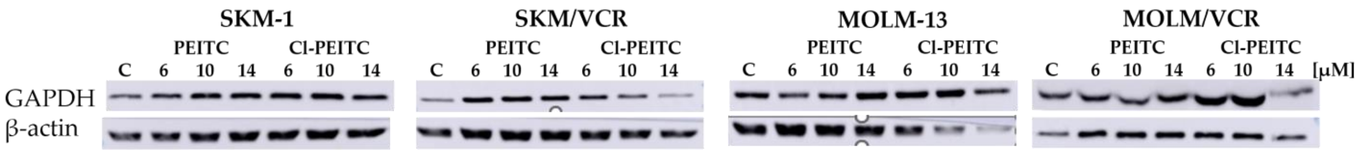

Figure S3. PEITC and Cl-PEITC affect the levels of typically used housekeeping proteins in human AML cell lines SKM-1, SKM/VCR, MOLM-13, and MOLM/VCR. Cells were exposed to PEITC or Cl-PEITC (6, 10, and 14 μM) for 12 h. Total protein extracts (60 μg/line) were determined for SDS-PAGE gel electrophoresis and subjected to immunoblot analysis using Anti-GAPDH (MAB374) and Anti-Actin N-terminal (A2103) antibodies.
